# Capillary Electrophoresis–Mass Spectrometry with Multisegment Injection and In-Capillary Preconcentration for High-Throughput and Sensitive Determination of Therapeutic Decapeptide Triptorelin in Pharmaceutical and Biological Matrices

**DOI:** 10.3390/biomedicines9101488

**Published:** 2021-10-16

**Authors:** Juraj Piešťanský, Ivana Čižmárová, Ondrej Štefánik, Michaela Matušková, Andrea Horniaková, Petra Majerová, Peter Mikuš

**Affiliations:** 1 Department of Pharmaceutical Analysis and Nuclear Pharmacy, Faculty of Pharmacy, Comenius University in Bratislava, Odbojarov 10, SK-832 32 Bratislava, Slovakia; piestansky@fpharm.uniba.sk (J.P.); svorcova7@uniba.sk (I.Č.); stefanik38@uniba.sk (O.Š.); matuskova53@uniba.sk (M.M.); horniakova24@uniba.sk (A.H.); 2 Institute of Neuroimmunology, Slovak Academy of Science, Dubravska cesta 9, SK-845 10 Bratislava, Slovakia; petra.majerova@savba.sk; 3 Toxicological and Antidoping Center, Faculty of Pharmacy, Comenius University in Bratislava, Odbojarov 10, SK-832 32 Bratislava, Slovakia

**Keywords:** capillary electrophoresis, tandem mass spectrometry, triptorelin peptide drug, field-enhanced sample injection, multisegment injection, pharmaceutical and biological samples

## Abstract

A capillary electrophoresis–tandem mass spectrometry method with a multisegment injection and an in-capillary field-enhanced sample stacking for determination of therapeutic peptide triptorelin in pharmaceutical and biological matrices was developed. The CE separation conditions were optimized in order to obtain maximal separation efficiency, analytical signal intensity and stability, and minimal adsorption of the analyzed peptide onto the capillary wall (1 M formic acid—HFo, pH 1.88). The implementation of the field-enhanced sample injection into CE improved the value of limit of detection 50 times while the multisegment injection increased the sample throughput three times in comparison to a conventional CE approach. The proposed method was characterized by favorable performance parameters, such as linearity (r^2^ ≥ 0.99), limit of detection (5 ng mL^−1^ in water matrix, 25 ng mL^−1^ in plasma matrix), precision (relative standard deviation, 1.5–9.4% for intraday and 2.3–11.9% for interday reproducibility), or accuracy (relative errors in the range of 80–109%). The FDA-validated method was successfully applied to the analysis of triptorelin in the commercial drug Diphereline^®^ 0.1 mg (powder for injection) and in spiked human plasma samples. Favorable performance parameters along with proven application potentialities indicate the usefulness of the proposed method for its routine use in drug quality control laboratories and for clinical analysis, such as determination of triptorelin levels in plasma (for pharmacokinetic study).

## 1. Introduction

Triptorelin is a synthetic decapeptide (pGlu-His-Trp-Ser-Tyr-D-Trp-Leu-Arg-Pro-Gly-NH2) used in the treatment of breast, endometrial and prostate cancer, and male hypersexuality with severe sexual deviation. The peptide is also used “off-label” as a puberty blocker in patients with gender dysphoria [1]. Triptorelin represents a synthetic analogue of gonadotropin-releasing hormone (GnRH) and is a modulator of the GnRH receptor where it can act as an agonist or antagonist. It is able to increase the circulating levels of luteinizing hormone, follicle stimulating hormone and testosterone. The stimulation of secretion of the above-mentioned hormones is responsible for growth-promotion which can lead to improvement of sport performances. According to these facts, triptorelin and other GnRH analogues were included in the Prohibited List of the World Anti-Doping Agency (WADA) and are summarized in Section S2 “Peptide hormones, growth factors, related substances, and mimetics” [2]. Therefore, there are demands for precise, accurate, robust, and sensitive analytical methods capable to identify and quantify triptorelin in pharmaceutical and biological matrices. 

Recent approaches for drug analysis are mainly based on liquid chromatography (LC) hyphenated with conventional (UV, DAD, fluorescence) or advanced (mass spectrometry, MS) detection techniques [3,4,5,6]. The LC strategies are considered as a gold standard for pharmaceutical and biomedical analyses. Although LC represents a dominating technique also for quantitative analysis of peptides and peptide or protein-based drugs [7,8,9,10], capillary electrophoresis (CE) is traditionally a powerful alternative to LC in analysis of biomolecules. This is due to several inherent benefits of CE including enhanced selectivity for charged substances, high speed of analysis, high separation efficiency and low consumption of the sample. Analogically to LC–MS, the coupling of CE with MS (CE–MS) significantly spreads possibilities in the analysis of complex biological mixtures [11,12]. 

There are only few papers which deal with the analysis of triptorelin by CE approaches. Those papers are focused on investigation of migration behavior of therapeutic peptide hormones [13,14], development of a CE–MS method for analysis of therapeutic peptide hormones including triptorelin [15], comparison of sheath-less and sheath-flow electrospray interfaces for CE–MS analysis of peptides [16], and development of a CE–UV method for study of the binding constants between an anionic polydispersed polymer and triptorelin relevant for development of the peptide drug delivery systems and their quality control [17]. All of these studies, however, were performed only with the use of model samples and peptide standards. The CE–MS methods for triptorelin determination [15] and [16] were characterized by the limit of detection (LOD) at 3.65 and 2.25 µg mL^−1^ levels and the reproducibility of the measurements (expressed as %RSD of peak area) at 15 and 33% levels, respectively. However, none of the previously published methods involved a complex validation protocol or application on real samples. 

An aim of the present study is to develop an effective and reliable CE–MS method for determination of triptorelin in real pharmaceutical and biological matrices. The proposed method should combine a simple in-capillary sample stacking procedure based on field-enhanced sample injection (FESI) with a multisegment injection (MSI) approach in order to achieve considerable enhancement in performance parameters. Optimization of the separation of electrolyte composition for preventing adsorption of the biomolecule onto capillary wall is an integral part of the method development as well. Improvements in sensitivity, reproducibility, accuracy, and sample throughput are highly demanded in routine control laboratories where the proposed method should be implemented.

## 2. Materials and Methods

### 2.1. Chemicals and Samples

LC–MS grade chemicals used for the preparation of the electrolyte solutions and sheath liquid were purchased from Merck (Darmstadt, Germany), Sigma Aldrich (Steinheim, Germany), and Fluka (Buchs, Switzerland). Demineralized water, prepared by a water purification system Millipore Simplicity 185 (UV) (Millipore, Molsheim, France), was used as a solvent for the electrolytes, sheath liquid, and samples. The electrolyte systems were filtered before use through disposable membrane filters (0.22 μm pore size Millipore) and were stored in the fridge before analysis. Triptorelin acetate was purchased from Caslo (Lyngby, Denmark) and the commercial drug Diphereline^®^ 0.1 mg (containing triptorelin acetate powder for injection) was obtained from Ipsen Pharma Biotech (Signes, France).

### 2.2. Instrumentation

The electrophoretic measurements were performed with the use of an Agilent 7100 capillary electrophoresis system (Agilent Technologies, Santa Clara, CA, USA). For CE–MS experiments an Agilent 6410 Series Triple Quadrupole tandem mass spectrometer (Agilent Technologies, Santa Clara, CA, USA) was coupled to the CE system by a commercial electrospray (ESI) sheath liquid interface equipped with a stainless-steel needle. The separation was performed in a 90 cm × 50 μm ID bare fused silica capillary (MicroSolv Technology Corporation, Eatontown, NJ, USA). The samples were injected hydrodynamically at 50 mbar for 20 s, unless otherwise stated. Experiments were conducted under normal polarity, applying voltage of 25 kV during electrophoretic separations. For CE–MS measurements the sheath liquid was delivered by an Agilent 1100 series isocratic LC pump and split to allow an effective flow from 1 to 10 μL min^−1^. All CE–MS experiments were performed in positive ion mode and in the Multiple Reaction Monitoring (MRM) mode. The dwell time was 200 ms.

### 2.3. Capillary Treatment

Prior to use, a new separation capillary was activated and conditioned flushing it for 15 min with aqueous 1M NaOH, followed by 15 min with demineralized water and 10 min with background electrolyte (BGE). All capillary rinses were performed at the pressure 950 mbar. Before each injection the capillary was re-equilibrated by applying a negative voltage of −20 kV for 30 s and flushing it with BGE for 2 min. At the end of each day, the capillary was rinsed with aqueous 0.1M NaOH and demineralized water for 20 min, with BGE for 10 min and stored in BGE overnight.

### 2.4. Field-Enhanced Sample Injection (FESI) and Multisegment Injection (MSI)

The FESI procedure consisted of the following steps: first, the capillary was filled with the BGE. Then a short plug of deionized water was hydrodynamically injected at 50 mbar for 5 s. Finally, a triptorelin sample was injected electrokinetically by applying a voltage of 10 kV for 20 s. 

The MSI was performed in CE according to following procedure: first, the capillary was preconditioned for 2 min by flushing with BGE. Then, a short plug of deionized water was hydrodynamically injected at 50 mbar for 5 s and the triptorelin sample was injected electrokinetically by applying a voltage of 10 kV for 20 s. After that, a BGE spacer plug was introduced at 50 mbar for 100 s. The procedure with sample and BGE spacer plug introduction was repeated three times. The experiments were conducted under normal polarity, applying voltage of 25 kV during electrophoretic separations.

### 2.5. Procedures for Sample and Standard Solution Preparation

A stock solution containing 1 mg mL^−1^ triptorelin was prepared by dissolving an appropriate amount of the triptorelin acetate standard in BGE. The working solutions of triptorelin were made by a proper dilution of the stock solution with demineralized water or plasma in glass vials. The concentration levels of triptorelin in the injected calibration solutions were in the range of 0.01–10 µg mL^−1^ (0.01, 0.05, 0.1, 0.5, 1, 2, 5, and 10 µg mL^−1^) for water and 0.05–10 µg mL^−1^ (0.05, 0.1, 0.5, 1, 2, 5, and 10 µg mL^−1^) for plasma. The pooled plasma samples were prepared by mixing a 100 µL volume of plasma from each individual plasma samples obtained from 5 healthy volunteers. The quality control (QC) plasma samples were prepared by spiking the pooled plasma samples (before protein precipitation) with the standard solution at three concentration levels: 0.05 µg mL^−1^ (low QC), 1 µg mL^−1^ (medium QC), and 10 µg mL^−1^ (high QC). A 10 µL aliquot of each QC sample was transferred into the Eppendorf tube and 30 µL of acetonitrile with 0.1% formic acid (HFo) was added. After 10 min at laboratory temperature, the sample was centrifuged at 13,000× *g* for 10 min. The supernatant was then transferred to a CE vial and directly injected into the CE analyzer. 

The pharmaceutical sample of triptorelin, Diphereline^®^ 0.1 mg (powder for injections), was prepared by reconstitution of the powder in the original ampoule with 1 mL of water for injections. The ampoule was shaken gently to form a homogenous solution. The stock sample solution was further 100-times diluted with demineralized water in a glass vial. The diluted sample was directly injected into the CE apparatus.

The plasma samples were obtained from five healthy volunteers. The study was conducted according to the guidelines of the Declaration of Helsinki and approved by the Ethical Committee of National Oncology Institute, Bratislava, Slovakia (protocol IZLO-1). The blood samples were collected in the morning in test tubes containing EDTA. Plasma was obtained by centrifugation (12,000× *g*, 10 min) within 30 min of sample collection. The samples were aliquoted and stored at –20 °C until further analysis. Before analysis, the plasma samples were left to thaw at 4 °C, and then 10 µL of each sample was transferred to an Eppendorf tube and 30 µL of acetonitrile with 0.1% HFo was added to precipitate proteins. After 20 min at laboratory temperature, the samples were centrifuged at 13,000× *g* for 10 min. The supernatant was then transferred to a CE vial and directly analyzed by the CE–MS method. Three consecutive injections of the sample were realized.

## 3. Results and Discussion

### 3.1. Optimization of CZE Separation

In general, the separation of various substances by the CE–MS combination demands volatile electrolytes with low ion strength [18]. Therefore, organic acids with low molecular weight, such as formic acid (HFo) or acetic acid (HAc), and their ammonia salts were tested as the BGE components. Analysis of therapeutic peptides by CE–MS is typically performed under acidic conditions [19] and this strategy was used also in our work. The effect of the BGE composition on the triptorelin signal intensity and stability is presented in Table 1. Higher concentrations of HFo were favorable for higher separation efficiency (expressed as number of theoretical plates, N) although slight increase of the migration time was also observed. On the other hand, the peak tailing occurred when the HFo solutions with lower concentrations were used for the separation. This may be caused by an unwanted adsorption of the peptide on the inner walls of the bare fused silica capillary. In the case of peptide separations, increased ion strengths are typically used to suppress adsorption [20,21,22]. In our work we demonstrated a 1000 mM HFo solution was effective for minimizing triptorelin adsorption (peak tailing was negligible). The improvement of peak shape was reflected also in an enhancement of peak area reproducibility (which was lower than 5% with a 1000 mM HFo solution). With respect to a maximum separation efficiency and reproducibility and a minimum adsorption of the analyte, a 1000 mM HFo solution (pH 1.88) was finally chosen as an optimum BGE. Under this acidic pH value, the silanol groups of the separation capillary wall are uncharged, and the electroosmotic flow (EOF) is eliminated. By eliminating EOF, only cations could migrate towards to detector. Thus, CE can serve as an effective ionic filter for anionic and neutral sample matrix constituents (i.e., prevent their detection interferences with the cationic analyte). Moreover, at the acid pH, the surface of the inner capillary wall is uncharged which is favorable for preventing adsorption of the analyte on it.

### 3.2. Optimization of MS Detection

#### 3.2.1. Electrospray Ionization (ESI) Step

The electrospray ionization (ESI) and MS detection steps were optimized to ensure proper identification and quantification of triptorelin. In the case of a commercial coaxial sheath-flow ESI interface, the composition of sheath liquid and its flow rate are the crucial parameters. 

The sheath liquid is responsible for appropriate ionization of the analytes and for establishing the required electrical contact between the liquid inside the separation CE capillary and the metal tube acting as electrode [23]. It is typically composed of an organic solvent mixed with a certain percentage of water and small amounts of volatile acid or base additives providing an enhancement of ESI efficiency. Here, two types of sheath liquids were investigated: (i) methanol/0.1% HFo water solution (50/50, *v/v*), and (ii) methanol/5 mM ammonium acetate (NH_4_Ac) water solution (50/50, *v/v*). The sheath liquid based on a NH_4_Ac additive was characterized by a sufficient stability of the electric current generated in the ionization chamber of the MS and an enhanced signal intensity of triptorelin, (with a 1.5-times higher S/N ratio) in comparison to this one based on a HFo additive. These findings were in a good agreement with our previous paper dealing with CE–MS analysis of immunogenic peptides [10]. Hence, the mixture of methanol with 5 mM NH_4_Ac (50/50, *v/v*) was finally selected as the optimum sheath liquid.

The sheath liquid flow rate is another important parameter affecting the effectiveness of the ionization procedure and thus stability and sensitivity of MS detection. Here, the sheath liquid flow rate in the range of 2–10 µL min^−1^ was investigated. An 8 µL min^−1^ sheath liquid flow rate was chosen as an optimum with respect to the highest S/N ratio along with a stable electrospray and analytical signal. 

Additional ESI parameters, responsible for an effective ionization procedure and stability of the analytical signal, were studied and optimized in the following ranges: nebulizing gas pressure (5–20 psi), drying gas temperature (150–350°C), drying gas flow rate (2–10 L min^−1^), and capillary voltage (3000–5500 V). The highest triptorelin signal intensity and stability were obtained for nebulizing gas pressure 10 psi, drying gas temperature 300 °C, drying gas flow rate 10 L min^−1^, and capillary voltage 5000 V.

#### 3.2.2. MS/MS Step

The optimization of MS/MS step included a chronological application of various triple quadrupole (QqQ) operation modes, namely Scan mode, Selected Ion Monitoring (SIM) mode, Product Ion mode, and Multiple Reaction Monitoring (MRM) mode. 

**Figure 1:**
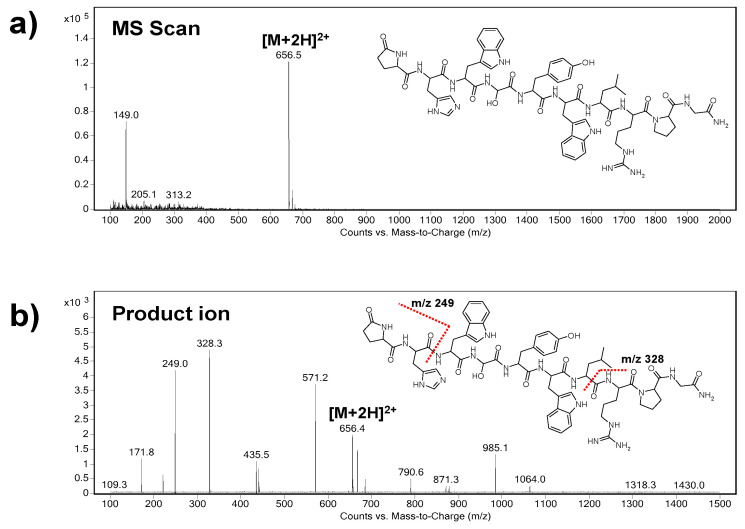
Representative parent ion (**a**) and product ion (**b**) triple quadrupole mass spectra of triptorelin. The spectral profiles indicate qualifier (*m/z* 249.0) and quantifier (*m/z* 328.3) product ions serving for unequivocal identification and quantification of triptorelin. The fragmentor voltage was set at 160 V and the collision energy was 20 eV.

At first, the precursor ion of triptorelin (*m/z* = 656.5) was indicated in the Scan mode (Figure 1a). As it can be seen, the *m/z* of the precursor ion represents double charged triptorelin ion. In the SIM mode, the fragmentor voltage in the range of 50–200 V was optimized. The highest intensity of the tiptorelin precursor ion was obtained when the fragmentor voltage was set at 160 V. 

Further, the collision cell energy in the range of 5–30 eV was optimized in the Product Ion mode in order to obtain characteristic fragmentation spectrum of triptorelin. The optimum collision energy, with nitrogen as collision gas, was 20 eV. Two most abundant characteristic ions, i.e., quantifier (ion with the highest intensity, *m/z* = 328.3) and qualifier (*m/z* = 249.0), were selected from the mass spectrum (Figure 1b). The selected product ions are in good agreement with the previous papers dealing with MS analyses of triptorelin [24,25]. 

Finally, the MRM mode was used for a highly reliable identification and quantification of the analyte. The following *m/z* ion transitions were applied: 656.5→328.3 (quantification transition), 656.5→249.0 (identity confirmation transition).

### 3.3. In-Capillary Sample Preconcentration and Improvement of the Sample Throughput

#### 3.3.1. Field-Enhanced Sample Injection (FESI)

**Figure 2:**
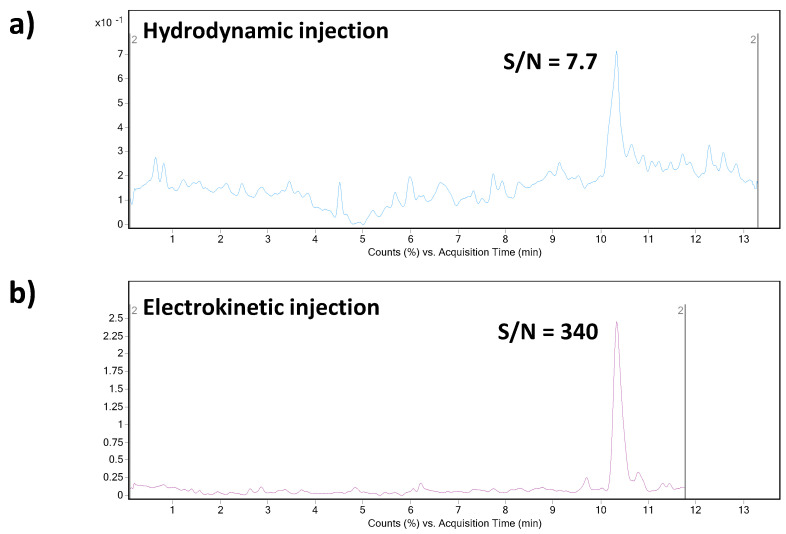
Effect of the CE injection mode (hydrodynamic vs. electrokinetic) on the analytical signal intensity. Concentration of triptorelin in the analyzed model sample was 0.5 µg mL^−1^. (**a**) Hydrodynamic injection was performed at 50 mbar for 20 s. (**b**) The electrokinetic injection (FESI) was performed at 10 kV for 20 s after the hydrodynamic introduction of a short water plug (50 mbar for 100 s) into the capillary. For other details of the CE–MS method see Section 2.

The above-mentioned previously published CE–MS methods dealing with the analysis of triptorelin suffered from relatively low sensitivity/high LODs and relatively poor peak area reproducibility (data in Table 2). In order to find conditions providing an enhancement of triptorelin LOD, we investigated and compared two approaches of the sample introduction into the separation capillary. The first one was represented by conventional hydrodynamic injection (50 mbar for 20 s). With our optimized CE–MS method, the LOD value for triptorelin was predicted at 0.25 µg mL^−1^. It is a ca. 9–15 times improvement in the LOD value when comparing to the published methods. However, this value is still insufficient for biomedical analyses (such as therapeutic drug monitoring or pharmacokinetics study) where triptorelin at ultratrace concentration levels is present. Therefore, an in-capillary sample preconcentration strategy based on a field-enhanced sample injection (FESI) technique was investigated for triptorelin as an advanced sample injection alternative. The FESI procedure was carried out by the introduction of a short water plug (50 mbar for 5 s) prior to the electrokinetic injection of the analyte. As it can be seen in Figure 2, the S/N ratio of the triptorelin sample at 0.5 µg mL^−1^ concentration level increased significantly when applying FESI. The data summarized in Table 2 clearly demonstrate benefits of the optimized FESI–CE–MS method over the published CE–MS methods for triptorelin in terms of obtainable LOD and reproducibility of measurements.

#### 3.3.2. Multisegment Injection (MSI)

The development of modern analytical methods aimed to a routine use is faced not only with the demands on a high sensitivity but also an enhanced sample throughput. One of the possibilities how to improve the sample throughput in CE is a serial injection of multiple sample segments within a single capillary. The multisegment injection (MSI) strategy performed in the CE–MS configuration has been predominantly applied in high-throughput metabolomics analyses [26,27,28,29,30,31,32]. Some papers deal with MSI–CE–MS also in drug quality control [33,34], screening of drugs of abuse [35], or peptide and protein analysis [36,37,38]. 

An MSI approach was implemented also in our analytical procedure. MSI is typically associated with the hydrodynamic injection of the samples. Here, we investigated the possibility to combine an in-capillary FESI preconcentration (based on electrokinetic injection of the sample) with a MSI procedure. The proposed MSI/FESI strategy was tested by three serial injections of the triptorelin model samples (prepared in water matrix). A baseline separation of three triptorelin peaks (corresponding to preconcentrated triptorelin zones migrating within three segments) was achieved when a short plug of the BGE (corresponding to hydrodynamic injection at 50 mbar for 100 s) was introduced into the CE capillary between each electrokinetic injection of the sample (i.e., FESI). The whole MSI/FESI–CE–MS analysis of three samples injected in one run was completed within 11 min, which corresponded to the analysis time of CE-MS with the conventional hydrodynamic injection of one sample. Thus, the presented data clearly demonstrated a 3-times higher sample throughput plus 50-times lower LOD for triptorelin with the proposed MSI/FESI–CE–MS method in comparison with the same CE–MS method employing conventional hydrodynamic injection.

### 3.4. Method Validation

The optimized MSI/FESI–CE–MS method was validated according to the ICH Q2(R1) and FDA guidelines [39,40]. Validation characteristics such as specificity, linearity, range, accuracy, precision, limit of detection (LOD), limit of quantitation (LOQ), and robustness were investigated. Measurements were carried out in water and plasma matrices. The evaluated validation parameters are summarized in Table 3 and Table 4 and discussed in the text below. 

Specificity, as the ability to assess unequivocally the analyte in the presence of other sample constituents, was demonstrated by the analysis of a blank saline solution and blank plasma spiked with the triptorelin standard at the LOQ concentration level. The sample matrices were chosen with respect to the intended application of the method. The results were compared with the data obtained from the analysis of corresponding non-spiked matrices. Thanks to a high orthogonality of the MSI/FESI–CE–MS/MS method (operating in MRM mode of MS/MS), no interfering compounds with the triptorelin peak at its migration time were observed in both saline and plasma matrices.

The calibration curves were established from eight calibration standards in the range of 0.01–10 µg mL^−1^ in model water samples and in the range of 0.05–10 µg mL^−1^ in pooled plasma samples. The calibration curves were expressed by the equation y = bx + a, where b represents slope and a represents intercept of the calibration line (for the data see Table 3). The parameters of the calibration lines were calculated with the use of Microsoft Excel 2007 (Microsoft Corporation, Redmond, WA, USA). Appropriate linearity (r^2^ > 0.99) in the range over two decadic orders was obtained for both matrices using the MSI/FESI–CE–MS method. The regression analysis indicated no statistical significance of the intercepts.

**Figure 3:**
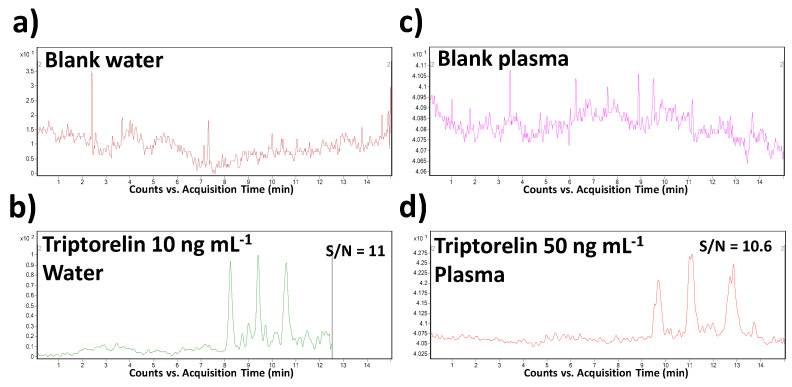
Representative multiple reaction monitoring (MRM) records obtained from the MSI/FESI–CE–MS analysis of (**a**) blank water (saline) sample, (**b**) triptorelin at the LOQ concentration level (10 ng mL^−1^) in water, (**c**) blank plasma sample, and (**d**) triptorelin at the LOQ concentration level (50 ng mL^−1^) in plasma. The records were obtained for the *m/z* transition 656.5→328.3. For other details of the CE–MS method see Section 2.

The LOD and LOQ values were calculated from the CE–MS profiles (of model water and plasma samples) as the signal-to-noise ratios (S/N) which should be 3:1 and 10:1, respectively. The predicted LOD values were 0.005 and 0.025 µg mL^−1^ in water and plasma matrices, respectively. When comparing with the literature, it represents the lowest level which was obtained for triptorelin in water matrix and the only LOD value determined and published for plasma by CE–MS methods. In comparison to the previously published CE–MS methods (in Table 2), a 450–750-times improvement of this parameter was reached. Illustrative records obtained from the MSI/FESI–CE–MS analysis of triptorelin at LOQ concentration levels in water and plasma are present in Figure 3.

A series of quality control (QC) triptorelin samples prepared in water and plasma matrices in the calibration range of 0.01–10 µg mL^−1^ was used to evaluate precision and accuracy of the developed MSI/FESI–CE–MS method (see data in Table 4). The precision was investigated as intra- and interday repeatability. The intraday precision was determined by measuring the samples (three consecutive runs) within one day. The interday precision was evaluated by repeated analysis of the samples (three replicates per day) for 4 days. The intraday precision (%RSD) ranged in the interval of 1.5–9.4% (water matrix) and 4.8–5.6% (plasma matrix). The corresponding accuracy (expressed as % relative error) was within the interval of 81–109% (water matrix) and 92.5–102.5% (plasma matrix). For the interday experiments, the precision varied from 2.3% to 11.9% (water matrix) and from 11.4% to 15.4% (plasma matrix) and the accuracy was within 80.2–109.4% (water matrix) and 93.6–96.6% (plasma matrix). The acceptation ICH and FDA criteria for precision and accuracy were accomplished so that the developed MSI/FESI–CE–MS method provides reliable quantification of triptorelin. 

The stability of triptorelin in pooled QC plasma samples was examined after storing them for 24 h in CE autosampler (short-term stability) and after performing three complete freeze and thaw cycles from −20 °C to +20 °C (freeze-to-thaw stability). The measured concentrations of triptorelin were in the range of 80.6–95.2% compared to the initial concentrations (Table 5). 

The suitability and reliability of the developed method for the demanded goal was also proven as robustness. Small and deliberate variations in method parameters, here variation of pH ( ± 0.1 unit) and BGE concentration ( ± 1 mM) were tested. No significant differences from the original (optimum) conditions were observed, the fluctuations of triptorelin migration time and peak area did not exceed 1%. Therefore, the optimized method is robust enough for its practical routine use.

### 3.5. Method Application

The optimized and validated MSI/FESI–CE–MS method was finally applied for quantitation of triptorelin in real pharmaceutical and biological matrices. 

**Figure 4:**
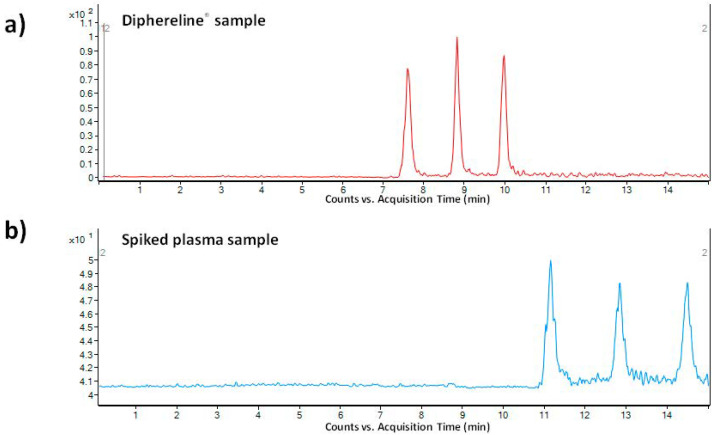
MSI-CE-MS analysis of (**a**) 100-times diluted pharmaceutical sample Diphereline^®^ 0.1 mg (powder for injection), and (**b**) plasma sample spiked with triptorelin standard at 0.1 µg mL^−1^ concentration level. The records were obtained for the *m/z* transition 656.5→328.3. For more details of the CE–MS method see Section 2.

As a pharmaceutical sample, a commercial drug Diphereline^®^ 0.1 mg (powder for injection) was analyzed. No extensive sample preparation before the analysis was necessary. A simple dilution with demineralized water was sufficient for this purpose. An illustrative record obtained from the analysis of a 100-times diluted drug dose is presented in Figure 4a. The declared content of triptorelin in the commercially available drug was 100 µg. The content of triptorelin determined by the proposed MSI/FESI–CE–MS method in three batches of Diphereline^®^ ranged in the interval of 97.67–101.18 µg, which was in good agreement with the value declared by the manufacturer. An excellent reproducibility of the measurements of pharmaceutical samples was demonstrated by the %RSD value which did not exceed 2.3% (*n* = 6). 

The application potential of the MSI/FESI–CE–MS method was demonstrated also via analysis of spiked human plasma samples at 0.1 µg mL^−1^ final concentration level of triptorelin (within the interval of triptorelin plasma levels in pharmacokinetics studies [41,42]). The preparation of model plasma sample was very simple. It included only protein precipitation (sample/acetonitrile with 0.1% HFo, *v/v* = 1:3) followed by centrifugation of the precipitated sample (13000× *g* for 10 min). The supernatant was directly injected. Ten spiked plasma samples (obtained from five healthy individuals) were analyzed in order to demonstrate an influence of the matrix variability on the fluctuation of electrokinetic injection and, by that, determined concentration of triptorelin. An illustrative record obtained from the analysis of triptorelin in a model plasma sample is presented in Figure 4b. Fluctuations of the concentration of triptorelin determined in ten different plasma samples ranged in the interval of 9.5–11.5%. Hence, the electrokinetic injection was demonstrated to be a highly reliable injection tool for triptorelin when combined with the MSI/FESI–CE–MS analysis method. The recovery (calculated as peak areas ratio of the analyte in the spiked pooled plasma sample with those measured at the equivalent concentration in a reference water matrix) of triptorelin from the pooled plasma sample, prepared by equimolar mixing of five different plasma samples, was 90%. These results clearly demonstrated usefulness of the developed method for highly effective and reliable monitoring of trace triptorelin in plasma samples.

### 3.6. Comparison of Methods for Triptorelin Analysis in Biological Samples

Table 6 provides a summary of chromatographic and electrophoretic methods used for determination of triptorelin in biological samples, i.e., plasma, serum, and urine. Determination of triptorelin has been typically performed with the use of liquid chromatography (LC) coupled with MS [25,42,43,44,45,46,47] or UV detection [48,49]. Actually, the only CE method developed for biological samples is this one presented in our work. Although the published LC procedures provided lower LOD values for triptorelin in plasma (0.006–6 ng.mL^−1^) than our CE method (25 ng mL^−1^), they needed an external sample preparation procedure (extraction) to be used for the selectivity and sensitivity enhancement. Thus, the developed CE–MS/MS method can be an advantageous alternative to the established LC–MS/MS approaches in terms of simplicity and cost. In addition, the LOD value obtainable by means of the developed CE–MS/MS method is more than suitable for investigating the first phases of triptorelin pharmacokinetics studies.

## 4. Conclusions

In the present study, a novel MSI/FESI–CE–MS method for quantitative analysis of triptorelin in pharmaceutical and biological samples was developed and validated. For the first time, the FESI in-capillary preconcentration procedure was implemented in the MSI strategy resulting in a considerable enhancement of both sensitivity and sample throughput. The method was applied for quality control of the commercial drug Diphereline^®^ 0.1mg. It was also successfully tested for the analysis of trace triptorelin in spiked plasma samples obtaining reliable analytical data in such variable multicomponent matrices. 

The complex validation procedure proved that the developed analytical strategy could be advantageously implemented in the field of quality control of triptorelin in pharmaceutical samples. Although the samples from patients treated with triptorelin were not available in this study, the validation data obtained from the analysis of a series of different plasma samples (obtained from healthy individuals) clearly demonstrated potentialities of the developed method for a routine monitoring of trace concentration levels of triptorelin in clinical samples (such as therapeutic drug monitoring, pharmacokinetic studies, compliance testing, etc.).

## Figures and Tables

**Table 1:** Optimization of the background electrolyte (BGE) composition of the CE–MS method for triptorelin determination.

BGE	pH	t_m_ (min)	RSD_tm_ (%)	RSD_area_ (%)	N	S/N
10 mM HFo	2.91	9.47	1.8	6.4	14,441	33.3
20 mM HFo	2.75	9.53	0.9	7.0	21,197	54.1
50 mM HFo	2.54	9.66	2.5	6.2	20,041	55.8
50 mM HFo + 50 mM HAc	2.85	10.42	0.5	20.7	24,552	52.5
1000 mM HFo	1.88	10.53	1.3	4.6	27,683	52.6
10 mM NH_4_Fo + 20 mM HFo	3.20	8.30	1.3	8.8	19,794	51.7

t_m_—migration time, RSD_tm_—relative standard deviation of migration time, RSD_area_—relative standard deviation of peak area, N—separation efficiency. S/N—signal-to-noise ratio. The concentration of triptorelin in the injected sample was 1 µg mL^−1^. The sample was introduced hydrodynamically at 50 mbar for 20 s.

**Table 2:** Comparison of selected validation parameters of the CE–MS methods for triptorelin in model water matrix.

Injection	RSD_area_ (%)	LOD (µg mL^−1^)	Reference
Hydrodynamical	15	3.65	[15]
Hydrodynamical	33	2.25	[16]
Hydrodynamical	8.9	0.25	This study
Electrokinetic	5.5	0.005	This study

**Table 3:** Operation and calibration parameters of the MSI/FESI–CE–MS/MS method for determination of triptorelin in model water and plasma samples.

Parameter	Water	Plasma
t_m_ (min)	10.53	14.54
RSD_tm_ (%), *n* = 6	0.24	0.99
RSD_area_ (%), *n* = 6	7.61	11.01
a (counts)	27.98	97.88
SD_a_, *n* = 6	1.12	11.99
b (counts.ng mL^−1^)	238.01	171.78
SD_b_, *n* = 6	2.52	4.65
r^2^	0.992	0.985
LOD (ng.mL^−1^)	5	25
LOQ (ng.mL^−1^)	10	50
*N*	36769	36956

t_m_—migration time, RSD_tm_—relative standard deviation of migration time, RSD_area_—relative standard deviation of peak area, a—intercept of the calibration curve, b—slope of the calibration curve, SD_a_ – standard deviation of intercept, SD_b_ – standard deviation of slope, LOD—limit of detection, LOQ—limit of quantification, N—separation efficiency. Separation efficiency (N) was calculated according to the equation N = 5.545 × (t_m_/w_1/2_)^2^. The separation efficiency was calculated for the analyte at its LOQ level.

**Table 4:** Accuracy and precision of the CE–MS/MS method for triptorelin in water/saline and plasma QC samples.

	Nominal (µg mL^−1^)	Found (µg mL^−1^)		RSD (%)		RE (%)	
Matrix		Water	Plasma	Water	Plasma	Water	Plasma
Intraday, *n* = 6	0.01	0.01	-	3.6	-	−19.0	-
	0.05	0.04	0.04	5.6	5.6	−10.0	−6.1
	0.1	0.11	-	9.4	-	9.0	-
	0.5	0.47	-	6.4	-	−5.8	-
	1	0.95	0.93	2.5	4.8	−4.6	−7.5
	2	1.95	-	2.8	-	−2.5	-
	5	5.24	-	5.5	-	4.8	-
	10	9.72	10.25	1.5	5.5	−2.8	2.5
Interday, *n* = 6	0.01	0.01	-	4.2	-	−19.8	-
	0.05	0.04	0.04	4.4	15.4	−14.2	−6.1
	0.1	0.11	-	11.9	-	9.3	-
	0.5	0.50	-	8.8	-	−0.1	-
	1	0.99	0.94	6.1	11.9	−1.5	−6.4
	2	1.93	-	4.0	-	−3.5	-
	5	5.47	-	5.3	-	9.4	-
	10	9.61	9.66	2.3	11.4	−3.9	−3.4

RSD—relative standard deviation, RE – relative error

**Table 5:** Stability testing of triptorelin in plasma QC samples.

	Autosampler Stability			Freeze-to-Thaw Stability		
	Low	Medium	High	Low	Medium	High
Nominal (µg mL^−1^)	0.05	1	10	0.05	1	10
Found (µg mL^−1^)	0.04	0.95	9.39	0.04	0.86	9.21
Accuracy (RE %)	−18.2	−4.8	−6.1	−19.4	−13.9	−7.9

**Table 6:** A summary of chromatographic and electrophoretic methods.

Method.	Study	Matrix	Sample preparation	t (min)	LOD (ng mL^−1^)	Ref.
LC–MS/MS	Pharmacokinetic study in rat	Plasma	Protein precipitation, solid phase extraction	~14	6	[42]
LC–MS/MS	Pharmacokinetic study in beagle dog	Plasma	Protein precipitation, solid phase extraction	~14	0.006	[43]
UHPLC–MS/MS	Monitoring of chemical castration	Serum	Protein precipitation, solid phase extraction	12	0.25	[44]
LC–MS/MS/MS	Pharmacokinetic study in rat	Plasma	Protein precipitation, micro-solid phase extraction	5	0.006	[45]
LC–MS/MS	Model study for antidoping	Urine	Centrifugation	15	0.25	[46]
LC–MS/MS	Pharmacokinetic study in rat	Plasma	Protein precipitation, solid phase extraction	6	0.006	[25]
LC–MS/MS	Pharmacokinetic study in rat	Plasma	Protein precipitation, solid phase extraction	3	0.3	[47]
LC–UV	Pharmacokinetic study in rabbit	Plasma	Dilution, electromembrane extraction	12	0.15	[48]
LC–UV	Model study of spiked human plasma	Plasma	Dilution, electromembrane extraction	40	0.6	[49]
CE–MS/MS	Model study of spiked human plasma	Plasma	Protein precipitation	15	25	This study

UHPLC—ultra high-performance liquid chromatography, MS/MS/MS – multiple mass spectrometry.

## Data Availability

Data is contained within the article.
